# Development of a DIPG Orthotopic Model in Mice Using an Implantable Guide-Screw System

**DOI:** 10.1371/journal.pone.0170501

**Published:** 2017-01-20

**Authors:** Miguel Marigil, Naiara Martinez-Velez, Pablo D. Domínguez, Miguel Angel Idoate, Enric Xipell, Ana Patiño-García, Marisol Gonzalez-Huarriz, Marc García-Moure, Marie-Pierre Junier, Hervé Chneiweiss, Elías El-Habr, Ricardo Diez-Valle, Sonia Tejada-Solís, Marta M. Alonso

**Affiliations:** 1 The Health Research Institute of Navarra (IDISNA), Pamplona, Spain; 2 Program in Solid Tumors and Biomarkers, Foundation for the Applied Medical Research, Pamplona, Spain; 3 Dpt of Neurosurgery, University Clinic of Navarra, Pamplona, Spain; 4 Dpt of Pediatrics, University Hospital of Navarra, Pamplona, Spain; 5 Dpt of Radiology, University Hospital of Navarra, Pamplona, Spain; 6 Dpt of Pathology, University Hospital of Navarra, Pamplona, Spain; 7 CNRS UMR8246, Inserm U1130, UPMC, Neuroscience Paris Seine - IBPS, Sorbonne Universities, Paris, France; University of Michigan Medical School, UNITED STATES

## Abstract

**Objective:**

In this work we set to develop and to validate a new in vivo frameless orthotopic Diffuse Intrinsic Pontine Glioma (DIPG) model based in the implantation of a guide-screw system.

**Methods:**

It consisted of a guide-screw also called bolt, a Hamilton syringe with a 26-gauge needle and an insulin-like 15-gauge needle. The guide screw is 2.6 mm in length and harbors a 0.5 mm central hole which accepts the needle of the Hamilton syringe avoiding a theoretical displacement during insertion. The guide-screw is fixed on the mouse skull according to the coordinates: 1mm right to and 0.8 mm posterior to lambda. To reach the pons the Hamilton syringe is adjusted to a 6.5 mm depth using a cuff that serves as a stopper. This system allows delivering not only cells but also any kind of intratumoral chemotherapy, antibodies or gene/viral therapies.

**Results:**

The guide-screw was successfully implanted in 10 immunodeficient mice and the animals were inoculated with DIPG human cell lines during the same anesthetic period. All the mice developed severe neurologic symptoms and had a median overall survival of 95 days ranging the time of death from 81 to 116 days. Histopathological analysis confirmed tumor into the pons in all animals confirming the validity of this model.

**Conclusion:**

Here we presented a reproducible and frameless DIPG model that allows for rapid evaluation of tumorigenicity and efficacy of chemotherapeutic or gene therapy products delivered intratumorally to the pons.

## Introduction

Diffuse Intrinsic Pontine Gliomas (DIPGs) represent the most frequent tumor among brainstem gliomas and constitute a real challenge for everyone devoted to the treatment of pediatric brain tumors[1,2]. Since its first description in the twentieth century, therapeutic alternatives remain scarce[3]. A dismal median overall survival between 9 to 13 months has remained unchanged in spite of combination of radiotherapy with targeted therapies. Unlike other brainstem tumors that benefit from surgical treatment such as focal pontine gliomas, exophytic, tectal or cervicomedullary tumors, DIPG due to its diffuse nature and anatomic extension within the pons, remains a fatal neoplasm[4]. Up to now the diagnosis was based on specific clinical symptoms and a characteristic radiographic appearance which usually shows a diffuse enlargement of the pons along with a variable and irregular contrast enhancement pattern. Thanks to the advent of biopsies which have led to an understanding of the genetic makeup of these tumors as well as generation of cell lines, several in vivo models have been recently developed including murine models that recapitulates the genotype of theses tumors[5–8] Most of these models employ stereotaxic-guided systems. The main advantage of using stereotaxy consists of precise access to the pons region throughout a biopsy needle aimed to a specific region in the brainstem according to previous standard coordinates. However, to perform studies with a high number of animals, such as survival studies with new therapeutic strategies or delivery of gene therapy agents, which need to be injected intratumorally, stereotaxy is extremely time-consuming, even in the hands of experienced researchers. In addition, if serial subsequent injections need to be performed there is the risk that they do not fall exactly in the same place.

In this work we set out to develop a rapid and reproducible DIPG model that recapitulates the histopathological features of diffuse pontine tumors but without the need of stereotaxic surgery. Previously, Lal et al described an implantable guide-screw system that allowed for rapid and consistent establishment of intracranial glioma xenografts that made consecutive intratumoral injection of potential therapies feasible [9]. Based on this approach we developed a DIPG xenograft that employs a guide-screw system inserted in the mice skull according to posterior fossa anatomic landmarks and directed to the pontine area. This model allows us not only for the generation of tumors in a fast and reproducible fashion but also to deliver therapeutic agents such as oncolytic viruses or immunomodulatory approaches, amongst others, through the screw fixed system.

## Materials and Methods

### Description of the guide-screw system

The guide-screw system was developed by the group of Dr. Fred Lang (UT MD Anderson Cancer Center, TX) [9] to study the effects of gene therapy on supratentorial intracranial gliomas xenografts avoiding the use of a stereotactic frame. Briefly, it consisted of a guide-screw also called bolt (#C212SG, Plastics One) a Hamilton syringe with a 26-gauge needle and an insulin-like 15-gauge needle. The guide screw is 2.6 mm in length and harbors a 0.5 mm central hole which accepts the needle of the Hamilton syringe avoiding a theoretical displacement during insertion. This system allows delivering not only cells but also any kind of intratumoral chemotherapy, antibodies or gene/viral therapy, etc.

The aim of this work was to develop a guide-screw based system specifically adapted to generate DIPG tumors without the use of a stereotactic frame.

### Establishment of DIPG coordinates

**Fig 1:**
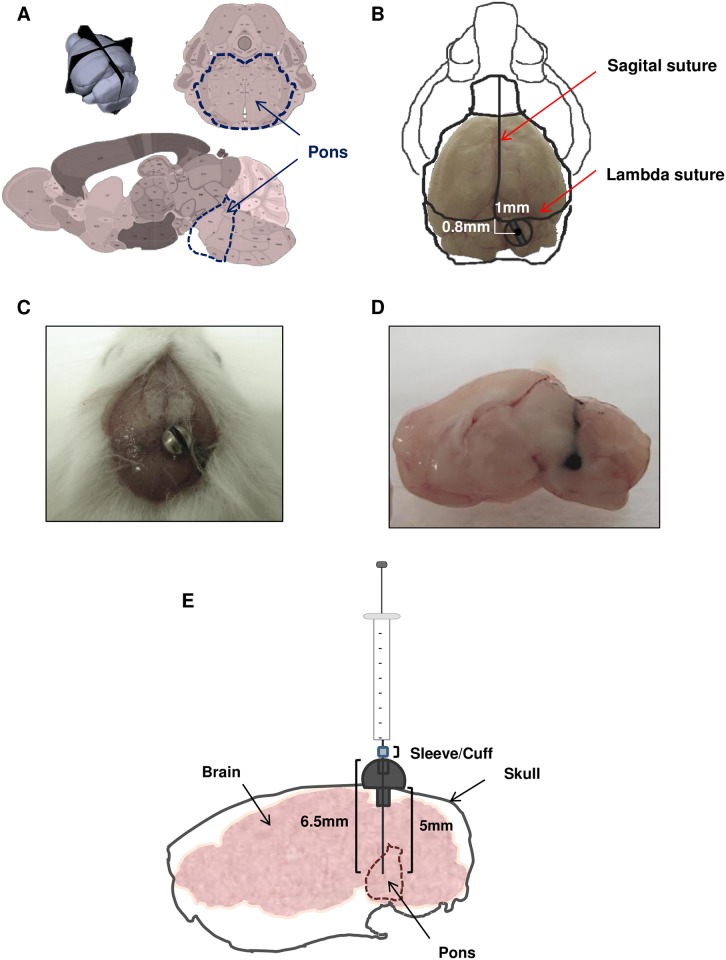
Screw guide system used to target the brainstem. A. Mouse Allen Brain Atlas scheme of brain structures, upper image is 3D image of mouse brain, left lower image is a sagittal scheme and right lower image represents a coronal image, bold dash line marks pons area in each image. B. Bolt coordinates in relation with lambda and sagittal sutures. C. Photo of mouse skull with bolt positioned in DIPG coordinates. D. Macroscopic photograph of ink solution injected at 6.5mm of depth with a Hamilton syringe through the bolt. E. Schematic drawing of screw guide components used in this administration method.

First, we set to establish the coordinates for generation of DIPG tumors using the Allen brain atlas. In addition, we use the Allen brain atlas to rule out the possibility that mice brain structures could be affected by Hamilton introduction (Fig 1A). We localize our entry point 1.0 mm right to lambda and just posterior (0.8 mm) to lambdoid suture (Fig 1B) so that large draining veins from posterior sinus can be avoided away from the target point [10].

Next, we assess the feasibility of the guide-screw placement using those coordinates and whether it would keep securely fixed over time (Fig 1C). We successfully inserted the guide-screw in the coordinates and it stayed secured for at least 30 days. Finally, it was necessary to establish the depth coordinate in order to generate the tumors in the right area inside of the pons. We injected an ink-based solution using a Hamilton syringe (26-gauge needle) fitted with a cuff at different depths ranging between 5-7mm (Fig 1D). This experiment allowed us to define the definitive coordinates to reach the pons: 1mm right to and 0.8 mm posterior to lambda as well as 6.5 mm depth insertion (Fig 1E).

### Technique for screw insertion

Under aseptic conditions and with all materials sterilized according to standard techniques, mice of four weeks of age were anesthetized by intraperitoneal injection with ketamine and xilacyne solution. The animal heads were supported by a couple of rolled gauzes so that when the screw was inserted, pressure applied over neck and head structures was better tolerated by the animal.

We prepared mice head skin with povidone iodine solution prior to make a 5 mm-long lineal skin incision with 23-size scalpel and expose both sagittal and lambdoid sutures. We first made a small mark according to the coordinates mentioned above with a small 15-gauge needle which was subsequently widened with a hand-controlled twist drill (Drill HSS, #8J60 Plastics One) which penetrates the skull until it reaches the duramater. At this point some bleeding from epidural space venous plexus could appear in some animals although constant pressure applied over the entry point with swabs should stop the bleeding. Next, we introduced the screw with its specific screwdriver by applying slight pressure throughout the previous twist hole until it was flushed with the cranial surface so that distal portion has protruded through duramater a few mm into brainstem area due to the particular configuration of brainstem in mice.

### Cell preparation and implantation

Once the screw was precisely inserted we proceeded with cell inoculation immediately after bolt insertion and during the same anesthetic period. We performed both procedures at the same time to reduce discomfort to the animal and to save time. Thereafter the needle of Hamilton syringe is slowly introduced into the hole by applying gentle pressure until the sleeve/cuff from the syringe reaches the screw surface and the desired depth (6.5 mm) is targeted. Roughly 10 minutes after bolt insertion, cell suspension (10^6^ cells in 3μl/per animal) was carefully injected using an infusion pump (Harvard Apparatus) over 20 minutes into the pontine region. With this technique the likelihood of reflux during cell administration was significantly reduced. At the end the needle was gently removed and the wound was closed with special surgical glue (Hystoacryl #1050044, Braum Surgical) so no sutures were required. Furthermore, recovery after surgical procedure is quicker and more comfortable because only one anesthetic dosage per animal was employed. At the end of the procedure all animals received an appropriate dosage of morphine solution as analgesic and waked up from anesthesia under warm conditions.

### Animal progression after surgery

Since animals were exposed to a highly invasive surgery they needed two days of analgesics. In addition we administered hydrant gels and gathered their food on the cage floor in order to facilitate food ingestion and accelerate mice recovery. Total mice recovery was achieved 72 hours after surgery with no visible symptoms caused by the procedure. Occasionally, some transient gait problems were observed in some animals during the first 48 hours after surgery that resolved spontaneously. No weigh loss was observed after surgery in any of the mice.

### Cell lines

For cell implantation we used the TP54 cell line derived from a biopsy of a patient with a DIPG tumor [11]. This cell line is characterize by a mutation in p53 (R248Q) a mutation in the K27M of H3F3A and is wild type for PTEN and ACVR1. Cell line were grown as neurospheres in a specific serum-free medium (NeuroCult^™^ NS-A Proliferation Kit, Human, #05751 Stem Cell Technologies) supplemented with EGF and bFGF in a humidified atmosphere of 5% CO_2_ at 37°C as previously described[11].

### Animal studies

Athymic mice were obtained from Harlan Laboratories (Barcelona; ES). Mice were maintained at the Centro de Investigación Medica Aplicada (CIMA; Pamplona; Spain) in specific pathogen-free conditions and fed standard laboratory chow. The study was approved by the committee of bioethics (CEEA; Comité Ético de Experimentación Animal under the protocol number CEEA/077-13). All animal studies were done in the veterinary facilities of the Center for Applied Medical Research in accordance with institutional, regional, and national laws and ethical guidelines for experimental animal care. The animals were monitored on daily basis and were euthanized when they demonstrate moribund behaviour including: slight head tilt, hemiparesis, hunched posture, scleral edema, inability to access food/water, weight loss >20% of baseline, and excessive tumor burden as indicated by doming of cranium >0.5 cm, or if show signs of lower extremity weakness. The animals were sacrificed with CO2 inhalation. To minimize suffering of the animals, ketamine/xylazine or buprenorphine was given for signs of pain, eye wincing, hunched state with front limbs over the head.

### Immunohistochemical analysis

Paraffin-embedded sections of mouse brains were immunostained with specific antibodies for H3K27M mutant (#ABE419 Millipore, 1:500), GFAP (Dako, Z0344 rabbit polyclonal, 1:500), human Nestin (#ABD69 Millipore, 1:500), Olig2 (#AB9610 Millipore, 1:500), Ki67 clone SP6 (Thermo Scientific, RM9106, 1:100) and human Vimentin clone V9, (M0725, Dako Denmark A/S, 1:400). Conventional procedures were followed in all cases.

## Results

### Tumor development, follow up and survival of mice bearing DIPG orthotopic xenografts

**Fig 2:**
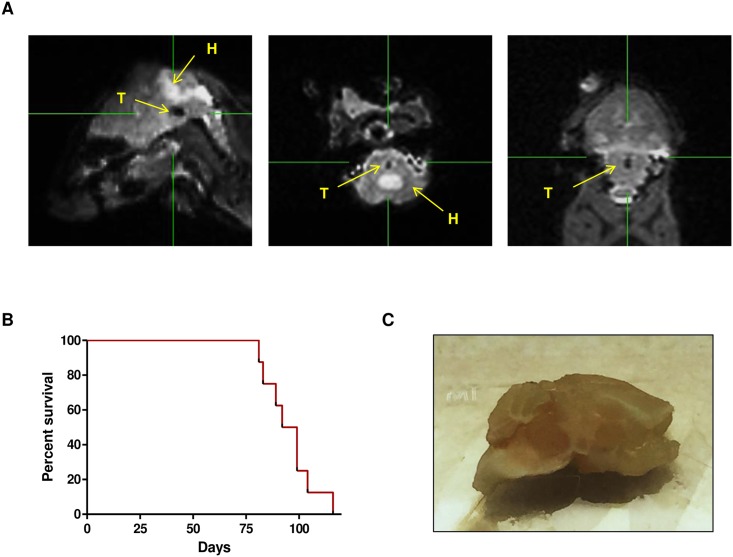
Tp54 tumor development in nude mice. A. Representative MRI of tumor development, left, central and right represent, respectively, sagittal, transverse and coronal views of a 3D T2-weighted sequence. Tumor (T) is seen as a hypointense dot in the pons, and hydrocephaly (H) caused by tumor pressure is seen as hyperintense dilatation of the mice ventricular system. B. Kaplan–Meier survival curve analysis for overall survival in athymic mice bearing DIPG xenografts tumors originated by engraftment of 10^6^ TP54 cells. C. Macroscopically image of mice brain with a visible tumor in the pons.

To validate the development of a frameless reproducible brainstem tumor model we injected the DIPG TP54 cells using our guide-screw system into the pons of nude mice (N = 10) according to the protocol described above. Animals were visually and physically checked for symptoms every two days during the first 4 weeks and daily beyond 30 days after surgical intervention. In addition, to rule out the possibility of brain damage magnetic resonance (3 Tesla MRI, Siemens Magnetom Trio and Magnetom Skyra) was performed to 5 random animals 15 days after surgery. MRI images showed no sign of damage or tumor at this early time point. We performed another MRI at a later time in these same animals when we observed symptoms compatible with tumor development in the pons such as animals spinning around themselves, showing gait disturbances and/or weight loss. In all these mice we found MRI images compatible with pontine tumors. In particular, in one of these animals besides the tumor (T) the images showed hydrocephaly (H) secondary to ventricular obstruction caused by the tumor[12] (Fig 2A).

Using the guide-screw engrafting technique 10 out of 10 mice developed tumors that resulted in progressive symptoms compatible with pons invasion. Animals bearing the TP54 cells in the pons had a median overall survival of 95 days ranging the time of death from 81 to 116 days (Fig 2B). Mice brains were extracted and tumors were detectable even macroscopically right into the core of the pons (Fig 2C).

### Pathological analyses of DIPG tumors

**Fig 3:**
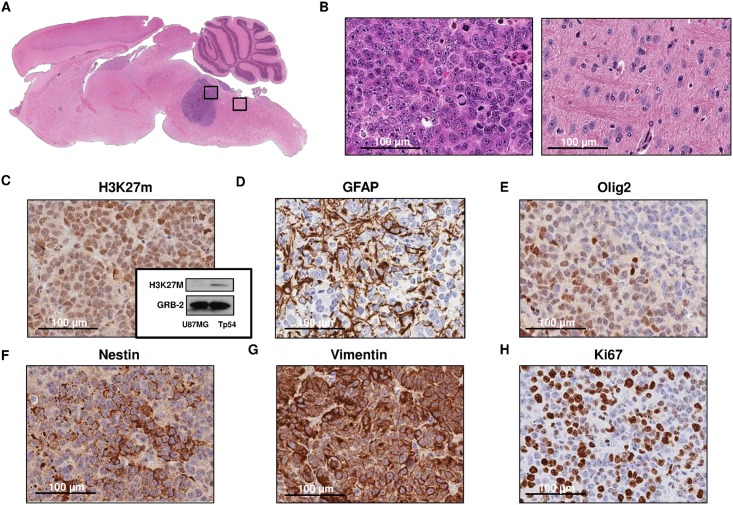
Pathological analyses of tumors developed by the TP54 cell line. A. Hematosilin-eosin stained of sagittal section of mice brain (x50) B. Right, tumor micrography image of hematosilin-eosin stained tumor section (400x). Left, detail of normal mouse brain (400x). Tp 54 tumor immunohistochemistry staining(400x): C. against Histone 3 mutation in lysine 27 and western blot, D. Glial Fibrillary Acid Protein (GFAP), E. Olig 2, F. Nestin, G. Vimentin and H. Ki67.

Next, we performed pathological analyses to characterize the tumors developed with our system. Hematoxylin–eosin staining of mice brains revealed that all tumors were localized in the pons (Fig 3A). Some of the tumors showed growth towards the cerebellar peduncles. The hematoxylin eosin staining showed a highly cellular and poorly differentiated tumor, composed by monotonous large and rounded cells with central nuclei and prominent nucleoli (Fig 3B). Several mitotic figures could be recognised. Vascular proliferation and necrosis were not evident in the tumor. Brain not affected by the tumor showed a normal morphology (Fig 3B).

Immunohistochemistry analyses showed positive staining for the H3K27 mutation, an aberration found in 80% of DIPGs tumors, in almost all cells (Fig 3C). Additionally, we analysed this mutation by western blot using the same antibody. As expected we corroborated the mutation in the H3K27M in the Tp54 cell line (Fig 3C). Tumor cells showed a glial phenotype as cytoplasmic expansions stained with GFAP. The nuclear staining against olig2 reinforces the glial character of the neoplasm (Fig 3D and 3E). It was previously described that these cells exhibited a neural profile expressing neural stem cells markers[11] including nestin and vimentin (Fig 3F and 3G). In addition, the tumor showed an intense proliferative activity according to the Ki67 immunoreactivity. Ki67 was found in 60 to 80% of the cells depending on the area evaluated (Fig 3H).

We have developed a reproducible and frameless DIPG model that allows for rapid evaluation of tumorigenicity, chemotherapeutic or gene therapy products delivered intratumorally to the pons.

## Discussion

Several years ago tissue sampling was not thought to be neither necessary nor suitable because of the potential mortality and morbidity associated with the biopsy process. The paucity of therapeutic alternatives led to redefine DIPG patients′ management in order to obtain a better understanding of the pathobiological pathways that would allow for more adequate treatment. Fortunately, the advent of technical breakthroughs, new biopsy protocols and interinstitutional collaborations has allowed a surge in research in this devastating disease including the development of several in vivo DIPG models [13–15].

Our group work is focused on the development of oncolytic viral and immune-therapies for pediatric brain tumors including DIPGs. Therefore, we needed a reproducible, fast technique that allowed not only for a reproducible engraftment of the cells in the pons but also for a system that facilitates the posterior delivery of different therapeutic agents into the tumor in the same area. Stereotaxy has proven as a secure and feasible system to develop preclinical DIPG models [5,6,16]. In fact, several groups have shown the validity of this technique to develop orthotopic DIPG tumors[2] that recapitulates the biology and phenotype of this disease. However, when there are a big number of animals per experiment due to a wide variety of treatment schedules or tested agents or the therapeutic agent needs to be delivered intratumorally stereotaxic technique, although precise, is extremely time-consuming. With this scenario in mind, the implantable guide-screw system developed by Dr. Lang at MD Anderson for brain tumor studies in small animals[9] provided the perfect system for the sequential delivery of different agents to the same anatomic region. Therefore, we used the guide-screw system to generate a DIPG model. A similar mode in rats was previously used by Hashizume et al [17]. In our study we adapted the system to be used in mice and we described the method in-depth for its feasible reproduction by other authors. The main advantage of employing this guide-screw system in the posterior coordinates that resemble children´s real DIPG location is the consistent cell delivery to the same area without the stereotaxic frame fact that saves a lot of time without compromising reproducibility and animal well-being. In addition, this procedure facilitates the delivery of therapeutic agents that are administered intratumorally, such as oncolytic adenoviruses, without the need of further surgery. As a result, this system allows for standardization of experiments when several groups are needed, facilitates tumor engraftment and the intratumorally delivery of different therapeutic agents in a reproducible and fast way.

## Conclusions

In this work we developed a preclinical in vivo DIPG model based on a guide-screw system fixed over mice skull that is feasible and allows for reproducible DIPG tumor generation in a fast and consistent fashion. This system permits the use of a considerable amount of animals for experiment and allows for the subsequent intratumoral injection of different therapeutic agents.
